# Fabrication and Applications of Magnetic Polymer Composites for Soft Robotics

**DOI:** 10.3390/mi14122173

**Published:** 2023-11-29

**Authors:** Sayan Ganguly, Shlomo Margel

**Affiliations:** Department of Chemistry, Bar-Ilan Institute for Nanotechnology and Advanced Materials (BINA), Bar-Ilan University, Ramat-Gan 5290002, Israel

**Keywords:** magnetic polymer composites, soft robotics, wearable gadgets, soft actuators, grippers

## Abstract

The emergence of magnetic polymer composites has had a transformative impact on the field of soft robotics. This overview will examine the various methods by which innovative materials can be synthesized and utilized. The advancement of soft robotic systems has been significantly enhanced by the utilization of magnetic polymer composites, which amalgamate the pliability of polymers with the reactivity of magnetic materials. This study extensively examines the production methodologies involved in dispersing magnetic particles within polymer matrices and controlling their spatial distribution. The objective is to gain insights into the strategies required to attain the desired mechanical and magnetic properties. Additionally, this study delves into the potential applications of these composites in the field of soft robotics, encompassing various devices such as soft actuators, grippers, and wearable gadgets. The study emphasizes the transformative capabilities of magnetic polymer composites, which offer a novel framework for the advancement of biocompatible, versatile soft robotic systems that utilize magnetic actuation.

## 1. Introduction

There has been a lot of interest in stimuli-responsive soft materials with static and dynamic shape programming and reconfiguration capabilities because they can easily integrate functions like actuation and sensing, which have promising applications in fields like soft robotics [1], reconfigurable structures [2], biomedical devices [3], morphable electronics [4], etc. The soft and compliant matrix may undergo massive and dynamic deformations, allowing for both active and passive physical adaptation to external environmental forces, stimuli, and restrictions, in contrast to traditional rigid materials [5,6,7]. Active fillers or microstructures in a variety of soft matrices allow stimulus-responsive materials to perform useful functions when exposed to the appropriate stimuli. Mechanical and linked multiphysical responses of the matrix for different physical behaviours and adjustable qualities can be induced by stimuli-responsive active components, such as micro/nanoscale particles, fibres, wires, polymer chains, or chemical groups [8].

**Figure 1:**
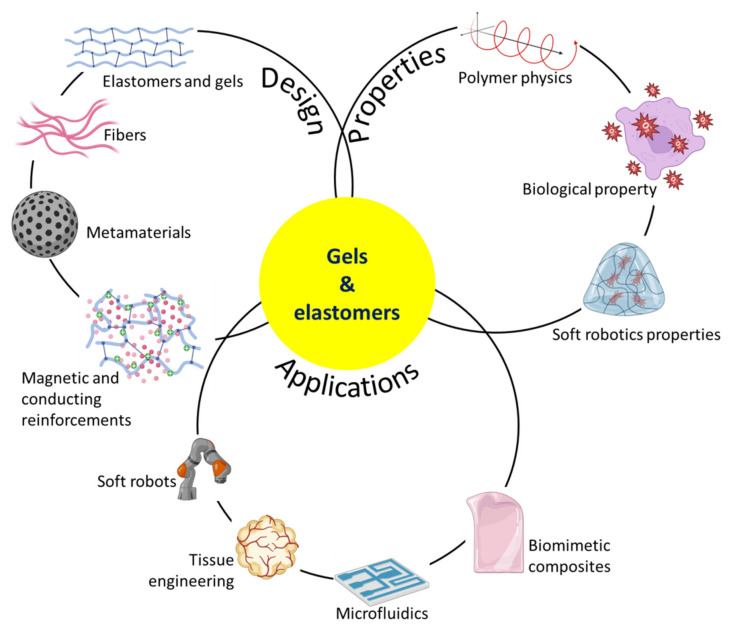
Schematic representation of the main things this review talks about. The review gives an overview of polymer physics principles, the properties needed for biomedical and soft robotics applications, the design strategies for reinforced elastomers and gels, and how they can be used in these areas.

Soft elastomers and gels reinforced with hard structures like woven/knitted fibres or 3D printed structures, frequently with conductive/magnetic components for active capabilities, have been widely used in biomedical and soft robotics research [9,10,11,12,13]. The use of variable stiffness technologies, meticulous structural design, and programmable actuation has led to the development of new classes of materials and robotic systems that facilitate more natural interactions between robots and people [14]. Although research has concentrated on developing structures with less stiff components [15,16,17], there are still downsides to using only soft materials. When it comes to supporting their weight, smaller organisms in nature might be softer, while larger species need a skeleton [18]. Therefore, in biomimetic and soft robotics applications, combining stiff structures with soft matrices is a promising technique [19,20]. Reinforced elastomers and gels, such as those seen in Figure 1, are discussed in this review for their usage in biomedical and soft robotics.

Since their mechanical properties are so comparable to those of soft tissues, elastomers (which can be either natural or synthetic) have long found utility in biomedical applications. Tough elastomeric gels are swollen polymers that may include solvents or physiological solutions and are thus called “elastomeric”. Since both are made of cross-linked networks of freely moving polymer chains, the same physical principles apply to interpreting their mechanical properties. The importance of soft tissues in biological function is a major inspiration for the field of soft robotics. The neurological and musculoskeletal systems of living things have had millions of years to coevolve with their environments, allowing them to accomplish tasks more efficiently and effectively together. We propose that the malleability and yielding nature of living things is crucial to the development of their embodied intelligence. Reinforcing with macroscopically structured hard materials, such as woven or knitted fabrics [21] or 3D printed structures [22], gives a feasible way to simulate natural tissue, whereas the toughness and rip resistance of elastomers and gels typically constitute a large challenge for biomedical application.

In order to provide fast, reversible actuation and locomotion in multiple degrees of freedom (DOF), magnetic soft composites, also known as magnetically responsive soft materials, can be magnetized from a distance [23]. The use of magnetically responsive soft materials has the potential to revolutionize soft robotics and the biomedical industries due to its outstanding performance in the construction of high-power density actuators [24]. Adjustable in strength, orientation, and coverage, magnetic fields can be used to precisely manipulate magnetically sensitive soft materials, allowing for the realization of many capabilities and greatly expanding the material systems’ potential applications [25]. The magnetic fields can also pass through many common substances, including air, water, and even living tissue. Due to their malleability, magnetic soft composites have the potential to revolutionize disciplines such as drug administration and minimally invasive surgery, where space is at a premium. Recent investigations have shown that diverse combinations of magnetic fillers, polymeric matrices, and fabrication methods can yield a wide range of functions [26,27,28]. The most recent developments in the material design and structure fabrication of diverse magnetic soft composites will be the primary focus of this discussion [29]. Once we have covered the various classes of magnetic soft composites, we shall focus on their most common features, such as the ability to change shape and adjust their properties, to use dynamic deformation for navigation, to manipulate and assemble objects, to produce heat and energy, and to use in reconfigurable electronics. We shall also discuss the wide variety of uses and accomplishments already attained by these materials. Finally, we shall talk briefly about the regulation of magnetic fields in general.

## 2. Magnetic Robot Based on Animal Designs

**Figure 2:**
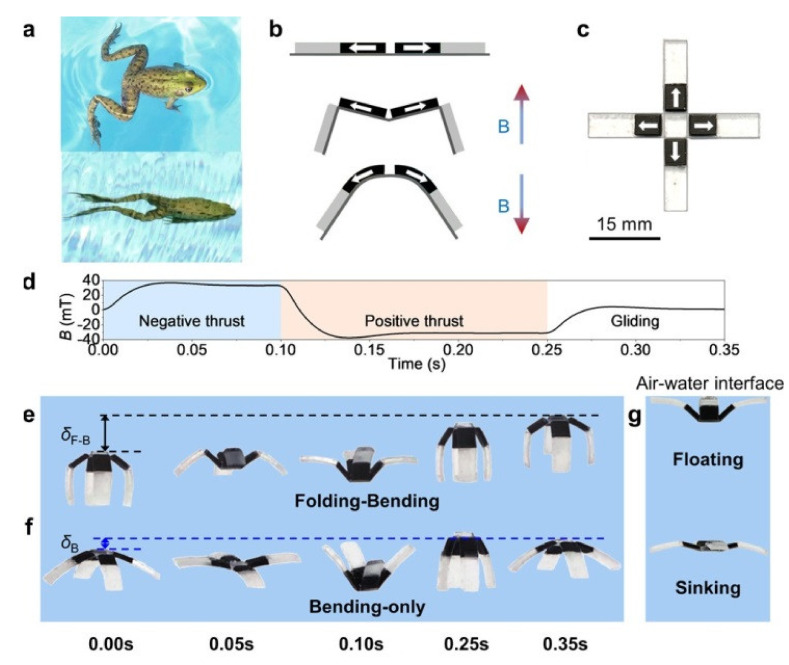
Making a biomimetic swimming robot and describing it. (**a**) How a moving frog moves forward; (**b**) a diagram showing the shape and preset magnetizations of a four-cell system; (**c**) built a swimming robot with four legs; (**d**) the controlling shape of the magnetic field; (**e**) the thrust performance during one turn of a magnetic field for a biomimetic folding–bending system with uneven joints; (**f**) a bending-only system; (**g**) floating and falling movements that can be controlled. Reproduced with permission from ref. [33]. © 2019 American Chemical Society.

The skeletal structure of soft-bodied robots has been heavily influenced by the varied modes of mobility found in animals. For example, arthropods like the inchworm walk with their characteristic circular motion, snakes move by swinging their bodies, and coelenterates like jellyfish swim by squirting and thrusting. The fundamental bell shape of a jellyfish’s body allows it to produce propulsion by squeezing and expelling high-pressure liquid at the cavity’s lower border. They can propel themselves, feed, and mix with the liquids around them by deftly manipulating the flow of fluids across their bodies while swimming at low energy, low noise, and high efficiency. At moderate Reynolds numbers, Ren et al. employed a magnetic elastomer fabrication technique to create a soft-bodied robot with magnetic control that mimics the morphology and mobility of jellyfish [30]. Like ephyra jellyfish, the swimming of bionic jellyfish is separated into a systolic phase and a recuperation phase: the systolic phase enables just the tail end of the tentacles to bend, while the recovery phase allows both the head and tail of the tentacles to bend. The robot moves with the help of a sheet-like magnetic composite elastomer driven by an external oscillating magnetic field, which, in conjunction with the two phases, causes a variety of regulated fluid flow phenomena around the body, allowing the robot to move forward. Researchers have studied the impact of this interaction on bionic jellyfish motion and have accomplished targeted grab and transportation based on the suggested lappet kinematics, which is the bionic jellyfish’s method of locomotion. More importantly, modelling bionic robotics’ unconstrained mobility on land after that of inchworms, polypods, and arthropods offers a suitable paradigm. Using permanent magnets embedded in silicone rubber, Niu et al. presented a centimetre-scale magnetic worm-like robot (MagWorm) [31,32]. The robot has excellent compactness and low cost; nonetheless, it travels inefficiently. Wu et al. developed a bionic magnetic robot using symmetry-breaking devices (Figure 2) to enhance the efficiency of its motion [33].

Footed robots that move like polypods, like ants and centipedes, have been described as a way to make movement even more efficient [34]. Centipedes and millipedes are two other robots that were inspired by multipedes and have been made to move over rough ground. Millipede-like robots move by having their legs move in a continuous metachronal wave motion that is triggered by a dynamic magnetic field and has a clear wave-like quality [35]. Likewise, travelling waves, a recurring mechanical deformation that comes from the way fish move, have become popular as a way to speed things up by rotating the magnetic head and causing changes in the nonmagnetic polymer tail [36].

## 3. Fabrication Methods of Magnetic Soft Robots

The manufacturing of magnetically sensitive materials is the topic of this subsection. Since the states of the materials are so different and the matrix materials used are so drastically different in chemical composition and properties, the preparation methods for magneto-responsive fluids/composites are also very different [37]. Preparation of MRFs requires careful attention to preventing oxidation and corrosion of the magnetic particle surface, as well as controlling the settling and agglomeration of magnetic particles using the features of the additives and the matrix itself.

Magneto-responsive composites require a more involved preparation process than other MRFs, involving steps beyond simple mixing, such as curing and magnetic particle orientation. Here we describe various popular techniques for making magneto-responsive composites.

## 4. Moulding

Spin coating, scratch coating, moulding, and so on are all examples of conventional fabrication methods. Since most of the magneto-responsive composites are matrix inlaid with magnetic particles and additional structures, they need to have a particular hardness to withstand deformation [38,39]. Therefore, its performance is heavily dependent on its thickness and rigidity. Moulding is preferable for magneto-responsive composites because it offers greater flexibility than the first two procedures. The advantages of moulding as a traditional method of composite manufacturing include its simple principle [40], ease of scheme design [41], material adaptability, and low manufacturing cost, all of which make it possible to achieve more complex spatial deformation through the design of moulds with varying shapes and the programming of magnetization at specific times during the curing process [42]. First, the polymer matrix is fully mixed with magnetic fillers and additives, and then it is laid into the mould and cured to make the desired shape or structure for the magnetic composite. Mouldable magneto-responsive composites can be made from a wide variety of polymer matrices, including hydrogels, elastomers, and SMPs [43,44,45]. Light curing, heat curing, dry curing, etc. can be used depending on the matrices. An external magnetic field applied to the magnetic particles during solidification can introduce anisotropic composites with a chain microstructure [46,47].

**Figure 3:**
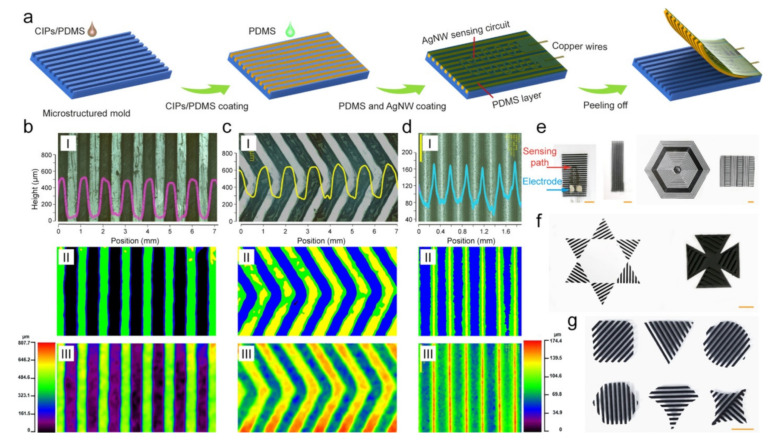
The process of fabrication and describing the structure. (**a**) Shows a schematic of the steps needed to make SMAF; (**b**–**d**) optical microscope pictures of MAF that is only structured on one side and has different microstructures (colour map (**I**), profile map (**II**), and height map (**III**)); (**e**–**g**) SMAF or MAF samples with magnetic material that is arranged in a certain way and comes in different sizes. The bar on the scale is 5 mm wide. Reproduced with permission from ref. [42]. © 2021 American Chemical Society.

The promise of shape-programmable magnetic materials and self-sensing conductive materials to realize functionality unreachable by conventional machines is appealing at small sizes. The magnetic materials and conductive materials for self-sensing magnetism-responsive anisotropic films (SMAF) can be predesigned, orientated, and patterned without the use of an external magnetic field generator or other costly devices, according to one study [42]. Magnetically powered flowers, windmills, and leaves are only a few examples of the novel shapes that have been made possible by magneto-active films with complicated chain-orientation patterns (Figure 3). Further investigation and development of aligned adaptable functions in the intelligent controlling structure and soft robotics is encouraged by the confirmation that as-prepared samples coated with the sensing layer can distinguish between actuation modes, like inward bending, outward bending, twisting, and combined deformation.

These complex microscopic structures can change shape at a large scale, and they are used in a lot of different ways in the field of soft robots. But the moulding cycle of the whole production process is slow and inefficient, and it is easy for bubbles to form when the material is injected into the mould. Also, when the products are taken out of the mould, the sides may have thicker flying edges. Some more advanced transformations and functions are currently not possible due to technical limitations.

## 5. Additive Manufacturing

More complex magnetic soft materials and robots with more nuanced actuation and functionality have been rapidly developed thanks to recent developments in additive manufacturing for soft materials. Extrusion-based 3D printing (for example, direct ink writing [48,49] and fused deposition modelling [50,51] and light-based 3D printing [52,53] are two examples of the additive manufacturing techniques used to create magnetic soft materials and robots of varying types (Table 1).

The tremendous progress of 3D printing technology in recent years has made it possible to not only build sophisticated surface patterns for magnetic materials but also to create programmable magnetic domains with a finer distribution. Using 3D printing technologies, researchers have shown that a wide variety of soft magnetic materials with acceptable structures may be created. Fused deposition modelling (FDM), direct ink writing (DIW), and vat photo-polymerization (VP) are introduced below as three example additive techniques. First, FDM technology is a type of 3D printing that relies on the extrusion of material. FDM technology has quickly risen in popularity and is now one of the most widely used additive manufacturing methods. The fused deposition modelling (FDM) procedure requires the fabrication of filamentary materials. In the production of filaments, it is customary to employ a conventional method of incorporating magnetic particles into the substrate material at a predefined ratio [61]. Poly (lactic acid) mixed with conductive iron (CI) filaments were 3D printed to create a variety of magnetic structural elements using FDM technology, which can produce fast reversible, programmable, and stable shape transformations, as proposed by Qi et al. [62]. Cao et al. introduced a novel magnetic actuator by the utilization of fused deposition modeling (FDM) technology for the fabrication of magnetic filaments composed of thermoplastic rubber and magnetic particles [63]. Rapid prototyping, a quick response time, and a programmable integrated structure are just a few of the benefits of FDM technology. However, applying this technology in practice is complicated, requiring steps like the fabrication of magnetic filaments before printing.

Fused deposition modelling (FDM) and direct ink writing (DIW) are two distinct techniques within the realm of 3D printing that share a common reliance on material extrusion. However, it is important to note that each method possesses its own unique limitations. Initially, it is worth noting that the resolution of the output is comparatively poor, which may result in the presence of additional imperfections or rough edges in the final outcome. Furthermore, the distribution of the magnetic filler can result in an unequal magnetic response [64]. In the field of expeditiously generating superior prototypes, VP stands out as a very sophisticated polymer manufacturing technology that is presently accessible. In the process of production, a printing chamber is utilized to contain a liquid resin that undergoes curing through light exposure. This curing process occurs selectively in predetermined regions. The magnetic particles exhibit enhanced affinity with the matrix and greater flexibility in terms of magnetization orientation due to the use of light-cured resin in the liquid state during the light-based 3D printing process. Stereolithography (SLA) and digital light processing (DLP) are two examples of induced-light polymerization techniques that use different types of light sources and scanning processes. In SLA, a laser is trained on the surface of the material, and the process continues from there, moving from point to line to surface. DLP’s most striking feature is its significantly faster printing speed compared to conventional SLA due to the curing process curing the entire coating at once rather than point by point [65]. Many researchers have turned to DLP in order to create magneto-responsive materials, such as those that can be printed with magnetic and nonmagnetic materials at the same time for easy and flexible assembly and connection of multiple parts [66]. The proposed approach incorporates a resin circulation system into a conventional VP platform, enabling continuous mixing of magnetic composites during the printing procedure. The purpose of this mechanism is to impede the rapid sedimentation of magnetic particles within the container during the printing process. This sedimentation, if left unchecked, may result in the unequal dispersion of fillers within the matrix or may possibly lead to printing failures [67]. This study also shows how the type of material and number of magnetic particles affect the VP process. Up to 30% of the resin is filled with strontium ferrite (SrFe_12_O_19_). For one-shot soft microrobotics, this method works well.

**Figure 4:**
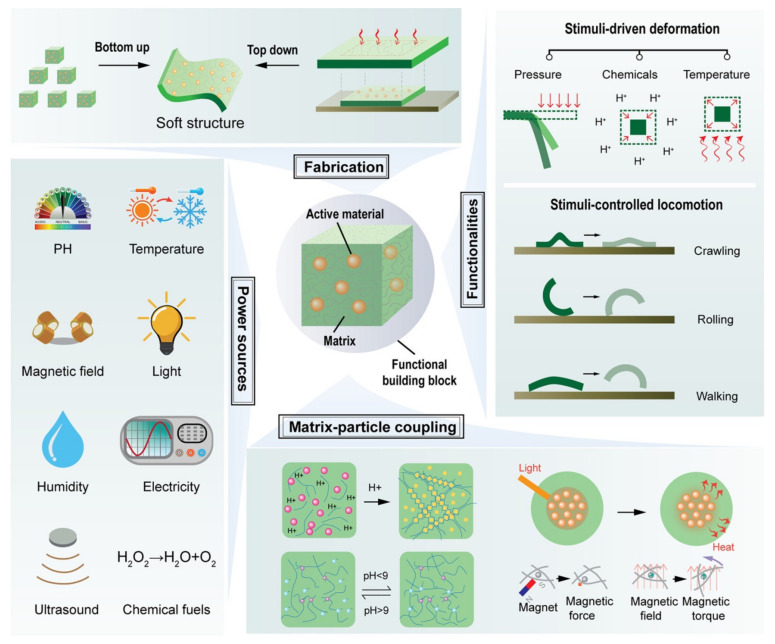
The state of the art in constructing responsive polymer–particle nanocomposites for use in creating soft and smart microrobots. Reproduced with permission from ref. [69]. © 2023 American Chemical Society.

AM makes it possible to make ceramic-based electronics with complicated 3D structures that can be given electronic functions in ways that have never been seen before. In one study, different AM technologies were used to make inductive force devices with a magnetic core made of ceramic and a helical spring made of polymer. We made sure the force sensing worked well by changing the spring constant of the helical spring and measuring the reaction to the outside force by looking at how the magnetic sensing circuit in the sensor changed its inductance (Figure 4). The fully additive manufactured (AMed) inductive force sensors worked very well at detecting [68].

## 6. Microfabrication

The above-described additive manufacturing processes have a shared characteristic of being limited by a feature size constraint typically measured in millimetres or centimetres. Various microfabrication technologies have been employed to manufacture magnetic soft materials on a small scale, typically ranging in diameters of tens or hundreds of micrometres. The utilization of UV photolithography has been employed in the construction of magnetic soft materials and robots at a small scale. These materials and robots are based on hydrogels made from poly(ethylene glycol) diacrylate (PEGDA) and contain superparamagnetic nanoparticles composed of iron oxide. The superparamagnetic nanoparticles are linked and aligned by an external magnetic field, while the selective cross-linking of the photocurable pregel solution is achieved through the patterning of UV light, either by masking or digital modulation. This process allows for the etching of desired magnetic or mechanical anisotropy patterns into the fabricated structures [57,58,59]. The ability to engage with micro- and nanoenvironments in a wireless and contamination-free manner is facilitated by microscale sensors and transducers that rely on magnetic forces. Nevertheless, the incorporation of magnetic components into conventional microfabrication techniques can pose challenges. The team of researchers has successfully constructed and described polymer micromagnets, which hold potential benefits for several fields like microelectromechanical systems (MEMS), microfluidics, microassembly, and microrobotics. These magnets are simple in design, cheap to make, and easy to print with conventional UV lithography [70]. They showed how to make polymer micromagnets with a feature resolution of 3 m and an aspect ratio of more than 10:1, how to move freestanding structures with the help of contactless applied magnetic fields, and how to make novel ‘hybrid’ magnetic microstructures with controlled heterogeneity of magnetic properties. Microcatheters, articulated microforceps, and tweezers with integrated sensing and actuation are in high demand because of the rise in the usage of minimally invasive surgery to treat minor lesions. The integration and functionalization of chemical and physical sensors present significant obstacles, yet existing microfabrication techniques have addressed their creation. Functionalization of fibres with diameters between 140 and 830 micrometres using a microrobotic platform has been described, with a patterning precision of 5 micrometres and an orientation error of less than 0.4 degrees. During a wet transfer operation, we were able to successfully align floating electronic circuits on a fibre thanks to two microrobots we built that measured 2 mm × 3 mm and had a thickness of 200 m. At the air–water interface, a permanent magnet was used to direct the movement and orientation of the microrobots. The average force applied was 0.5 N, with 0.2 newton millimetres of stiffness associated with the position being regulated [71]. The distance between the two microrobots could be precisely adjusted due to the magnet’s nonhomogeneous magnetic field and the microrobots’ differing preferred magnetization directions. One of the microrobots was programmed to act as a tweezer, grabbing and releasing free-floating electronic patterns, while the other microrobots were employed to align the pattern’s position and orientation with the fibre. Likewise, a model for the microrobots’ surface-tension-based interaction is proposed. Microstructures that are powered and controlled wirelessly in fluid environments are crucial for applications where contamination must be avoided, such as cell manipulation, and for applications where connecting the power source to the actuator would be cumbersome, such as targeted delivery of chemicals [72]. A novel production methodology was suggested to enclose magnetic composite materials within pure SU-8 structures, hence facilitating the development of microscale ferromagnetic microrobots that possess both biocompatibility and chemical resistance. The proposed microrobots are simple enough for mass production thanks to multilayer photolithography; they are activated contact-free by an external magnetic field and are used for performing micromanipulations. In order to move a glass microsphere along a predetermined course at a speed of 1.1 mm/sec, the microrobots were activated to spin, aim at targets, and collect things along the way. Biological samples and living organisms are delicate; therefore, conventional stiff systems cannot handle them. Also, the submillimetre range and above size of rigid systems prevents them from being used in biological scenarios involving even smaller cavities, such as blood arteries and intracellular spaces [73,74]. Many researchers have investigated active nanomaterials in the presence of soft (polymeric) matrices and external stimuli, such as light, heat, electric field, and magnetic field, in an effort to overcome the aforementioned obstacles [75,76]. However, the microassembly method based on micromoulding, magnetization, and human assembly of the building blocks to construct 3D things can be labour-intensive and, thus, is probably not scalable without an automated workflow [77]. The development of automated fabrication techniques will play a crucial role in advancing the research and development of next-generation magnetic soft robots. These robots are expected to possess enhanced functionalities and sophistication. Specifically, the focus will be on realizing small-scale magnetic soft materials and robots in fully three-dimensional (3D) shapes, accompanied by intricate 3D magnetization patterns. This will enable the achievement of complex transformations in these robots [78].

## 7. Applications of Magnetic Robots

Soft magnetic microrobots can be used in living things and have many benefits, including being able to be controlled remotely, rearranged, programmed, and recycled. They have a wide range of uses in biological engineering. In this part, we talk about how magnetic microrobots can be used in biomedical areas, such as for minimally invasive surgery, in vivo detection, and moving small loads.

### 7.1. Cargo/Payload Transport

**Figure 5:**
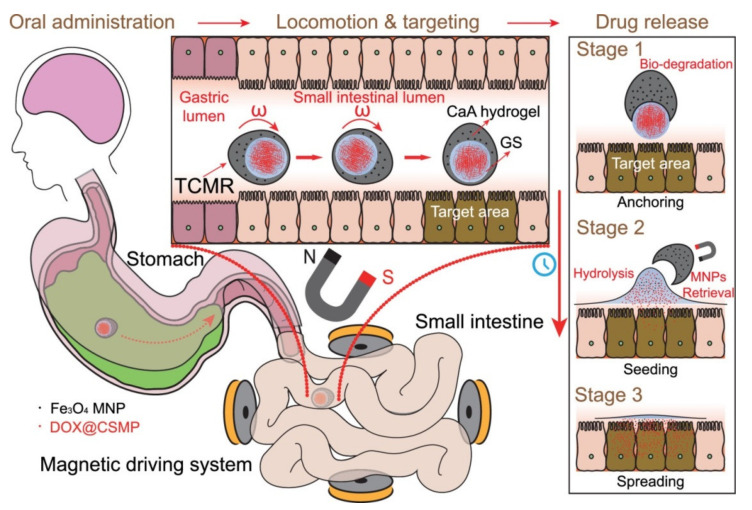
Oral delivery of TCMR for targeted therapy in the small intestine is depicted in this schematic. Reproduced with permission from ref. [82]. © 2021 American Chemical Society.

**Figure 6:**
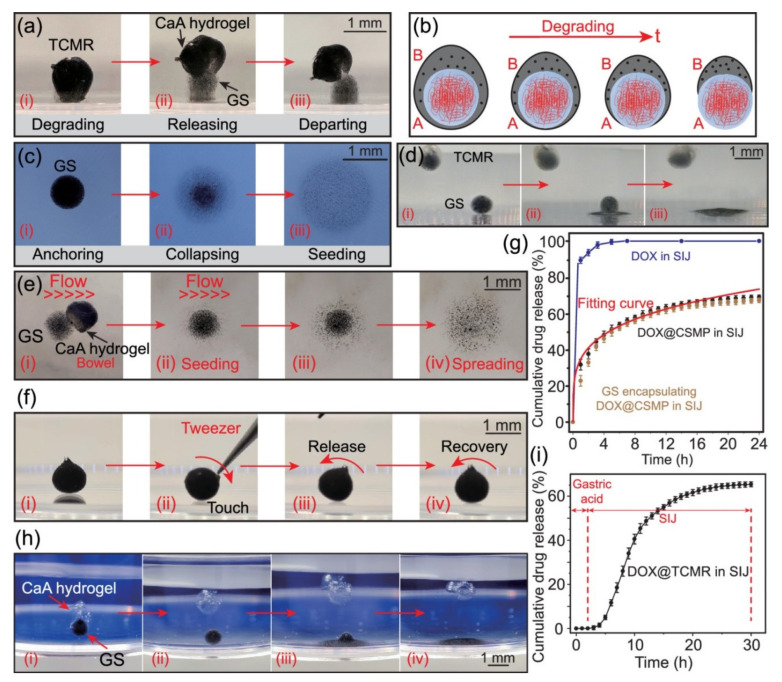
The ways that TCMR releases drugs. (**a**) Graphs showing the order of events during the first stage of release (GS release). (**i**) TCMR breaking down; (**ii**) GS getting free from TCMR; (**iii**) TCMR splitting into two parts. (**b**) The TCMR changed during the first stage of release. (**c**,**d**) The second stage of release, called GS hydrolysis, is shown in chronological order in the images. (**i**) GS attaching to the base; (**ii**) GS breaking down when water hits it; (**iii**) GS starting the CSMPs. The time-sequenced diagrams (**i**)–(**iv**) show how pure GS and GS wrapped in TCMR behave in the SIJ setting. (**e**) The GS fixes the goal position so that a flow of SIJ can seed CSMPs. (**f**) The TCMR staying in the right position at the target spot. (**g**) If you heat the CSMPs to 37 °C, they will release a total of % DOX. (**h**) This is the whole process of DOX releasing from TCMR (without Fe_3_O_4_ MNP). (**i**) The total amount of DOX released (in %) from TCMR in the artificial stomach juice and the SIJ one after the other. Reproduced with permission from ref. [82]. © 2021 American Chemical Society.

As a replacement for the conventional approach, research has been conducted into the use of wireless magnetic robots and their related magnetic navigation systems (MNSs). Multiple scientists have investigated MNSs. Choi et al. built an MNS to operate a magnetic helical robot (HR) using four sets of coils: two sets of Helmholtz coils and two sets of Maxwell coils [79]. Researchers have looked into the movement of a miniature robot for use in intravascular treatments. Because of the need for portability, an intravascular microrobot cannot have the same components as a regular robot. To address this confluence issue, we conducted a thorough analysis of an electromagnetic actuation (EMA)-equipped microrobot. The EMA system included four coils, two in the Helmholtz direction (x) and two in the Maxwell direction (y). Two sets of Helmholtz coils and one set of Maxwell coils are proposed in this research as part of a revolutionary stationary EMA system. Experiments are conducted to test and validate the proposed EMA system and its application to the microrobot’s 2D locomotion. The microrobot powered by the suggested EMA system travelled on the planned trajectory. To achieve the same level of actuation force, the suggested EMA system used 91% less coil current and occupied 18% less space than the prior EMA system. Investigations have also been conducted into a wide variety of magnetic robots, all of which are controlled by the MNSs. These magnetic robots include capsules, crawling robots, inchworms, earthworms, and HRs [80]. A tetherless soft capsule endoscope with a magnetically triggered multimodal medication release mechanism was presented by Yim et al. for the treatment of gastric illness. The medicine might be released locally at a targeted location if the constructed capsule, which contains a drug chamber between its two magnetic heads, were compressed by an external magnetic field. The medicine can be released in one of two ways from the capsule, depending on the circumstances. The first setting uses a series of pulses (0.01–0.03 T) of magnetic field to gradually release a modest dose of medication. The frequency of an external magnetic pulse was shown to regulate drug release in laboratory experiments. In the second mode, an external magnetic field of 0.07 T creates a magnetic attraction higher than the critical force for the capsule to collapse, resulting in the release of approximately 800 mm^3^ of drug. This results in the capsule being encased in a polymeric sheath. The coated surface area is a function of the drug’s viscosity. The creeping microrobot presented by Nam et al. can successfully traverse and anchor in a variety of tubular habitats, such as human blood arteries and pipes. The microrobot has a flexible body, magnetic bodies that can rotate separately, and connecting rods and legs [81]. The differential force of friction at the location of contact with the leg and the wall of the tube makes it resistant to the forces of gravity and fluidic drag. Controlling the currents in a magnetic navigation system (MNS) allows for forward and reverses crawling movements of the microrobot, as the fluctuating magnetic field produced by the MNS can induce oscillating motion of the microrobot. This research also presents a mechanism that efficiently produces a three-dimensional (3D) pulsating magnetic field for the exact operation of the microrobot in a 3D cylindrical environment. There has been a lot of interest in using active targeted therapy with untethered microrobots for bowel cancer. Problems with mobility, biocompatibility, drug loading, sustained-release capabilities, and targeting accuracy plague conventional microrobots. In their paper, Chen et al. presented a tethered magnetic robot with three nested configurations: actuation and guarding, anchoring and seeding, and drug release [82]. Using a targeted magnetic drive mechanism, the triple-configurational magnetic robot is actuated to follow the planned path to the intended destination (Figure 5). Electrodeposition forms a pH-sensitive actuation and guarding component, which degrades in the acidic intestinal environment and breaks apart. The vast bulk of magnetic nanoparticles are recovered from this section. Hydrolysis of the anchoring and planting portion plants the drug release portion in the villus sulci. The sustained release of the therapeutic agent is completed by the drug release component containing the therapeutic agent. Triple-configurational magnetic robots have been shown to be biocompatible, appropriate for targeted therapy, and to have high therapeutic performance in cytotoxicity and therapeutic testing (Figure 6). The newly developed triple-configurational magnetic robot will contribute to the advancement of tailored therapy, broadening the range of potential robotics applications in the biomedical sector.

**Figure 7:**
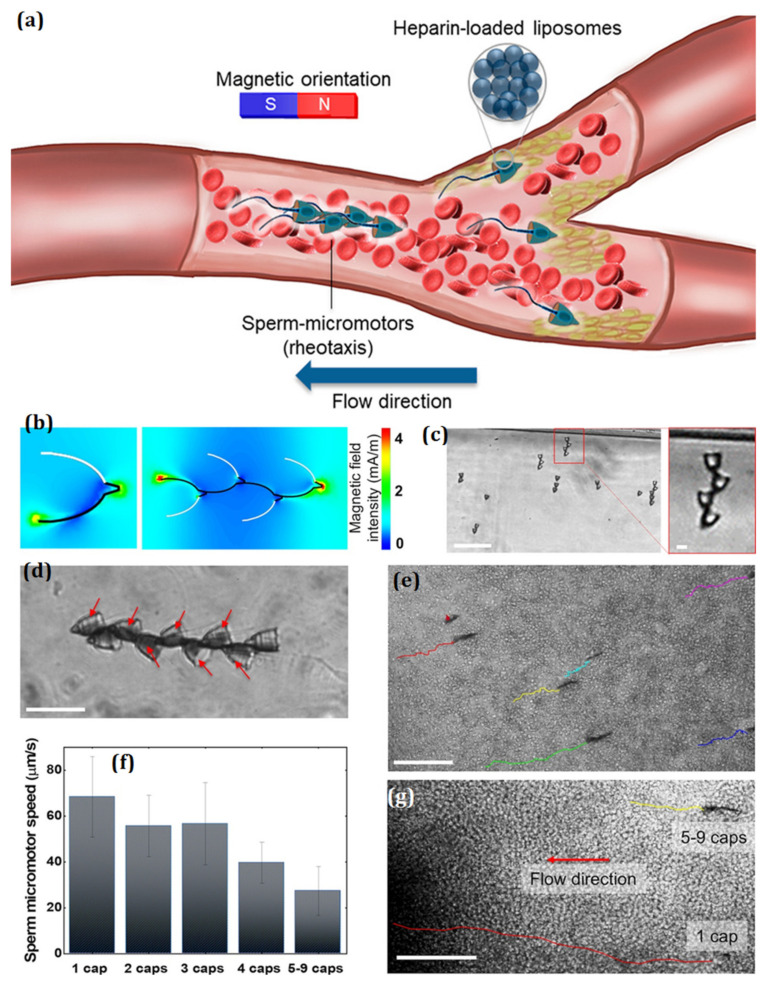
(**a**) Concept of the blood-adapted SHC sperm micromotors. (**b**) Magnetization models of a SHC sperm train and a 4-SHC sperm train, both of which had some iron on them. The part that is covered in metal is black in colour. Alignment of SHCs that have been put together without sperm. (**c**) Sperm train swimming in sperm medium (SP-TALP). The heads of the sperm are shown by red lines. (**d**) A group of sperm trains moving in blood that has been diluted by four. (**e**) The number of caps matches the swimming speed of sperm trains (n = 6). (**f**) A sperm train track and a SHC sperm micromotor moving against the flow of blood. (**g**) The lines on the graph show how much something weighs. Reproduced with permission from ref. [86]. © 2020 American Chemical Society.

Researchers have fabricated magnetic robots in the past. Microrobots that look like insects are used in many biomedical tasks. For example, experts have made inchworm microrobots that can be used for endoscopic tasks. A biological inchworm moves by twisting its legs. But most inchworm microrobots depend on a move called “bellows,” which means they bend. To achieve a looping gait, a new robotic mechanism is introduced here that regulates magnetic force and torque in a revolving magnetic field. The magnetic torque, attraction force, and body mechanisms (two stoppers, a flexible body, and several frictional legs) are all under the control of the suggested robot [83]. A bending motion is produced by magnetic torque. Without an algorithm for field control, the robot’s attraction force and body mechanisms produce its looping motion within a spinning magnetic field. When compared to permanent magnets, the shape, flexibility, ease of manufacture, and combinative ability of the flexible magnetic actuator (FMA) made from Sm–Fe–N magnetic silicone rubber (MSR) is much greater. To test its potential for usage in an FMA, we studied its magnetic and elastic properties and concentrated on magnetic torque control inside a uniform rotating magnetic field [84]. After combining Sm–Fe–N powder with silicone rubber liquid and hardener, the resulting mixture was poured into a mould. The engineered Sm–Fe–N MSR had Sm–Fe–N powder with various vol% ratios (13.5, 17.3, and 21.2). A vibrating sample magnetometer (VSM) and an elastic load were used to analyse the material’s physical and elastic properties. To boost a microrobot’s movement and drilling capabilities in a two-dimensional environment, Jeong et al. presented a modified EMA system. A total of four sets of coils—three Helmholtz and one Maxwell—make up the proposed EMA system. The microrobot we used in this investigation also differs structurally from the aforementioned microrobots in that it has a spiral-shaped body and is made up of two magnets with opposite magnetization [85]. A fusion algorithm between the precessional magnetic field and the gradient magnetic field is developed for use in the actuation process. Finally, we demonstrate the viability of the microrobot using the proposed EMA system for medical application through a series of tests in which we evaluate the improvement of locomotive and drilling performances produced by the system. Untied micromanipulation and targeted cargo delivery in complex biological contexts are two areas where micromotors have been identified as having great potential. Although synthetic micromotors have been described for use in the circulatory system, their low thrust force has prevented them from being widely implemented since it is insufficient to overcome the high flow and complicated composition of blood [86]. The hybrid sperm micromotor reported by Xu et al. is capable of actively swimming against the flow of blood (both steady and pulsatile) and delivering heparin (Figure 7). The synthetic microstructure provides magnetic guiding and cargo conveyance, while the sperm flagellum delivers tremendous propulsion force. In addition, after being magnetized, individual sperm micromotors can form a train-like carrier that can carry many sperm or medical cargoes to the target location; these could one day be used as anticoagulant medicines to treat blood clots and other circulatory ailments.

### 7.2. Magnetic Robots in In Vivo Imaging

Biomedical micro- and nano-scale robots’ controllability, aesthetic appeal, usefulness, and biocompatibility are crucial design considerations. Smaller robots, such as micro- and nano-scale robots, may have trouble moving in fluid environments because the Reynolds number is so low compared to that of air. In the low Reynolds number regime, inertia is small relative to the viscous force, hence unique locomotion strategies must be created to actuate and steer the tiny robots. Artificial micro/nanoagents are typically subdivided into self-propelled and external-field-propelled varieties, while natural/biological, artificial, and biohybrid varieties of micro/nanorobots can be broadly categorized into these three broad classes [87]. Micro- and nanorobots that move themselves typically acquire power from their surroundings in the form of self-electrophoresis, self-thermophoresis, self-diffusiophoresis, and tiny bubbles. External-field-driven micro- or nanorobots, on the other hand, can only move if an external field is applied, such as a magnetic field, an electric field, light, US waves, or something else. Real-time imaging and monitoring of micro- and nanorobots is essential for their use in biomedical applications, especially in vivo. Fluorescent imaging (FI), MRI, US imaging, computed tomography (CT), positron emission tomography (PET), and single-photon emission computed tomography (SPECT) are just some of the imaging modalities that have been investigated for localizing micro- and nano-scale robots. The resulting micro- and nano-scale robots can be tracked in real time in vitro and even in vivo, and they may be guided to their intended locations for targeted distribution and therapy using vision-based control. When compared to micro-/nanoagents that remain stationary, the added motion introduced by these agents may also improve imaging contrast due to the dynamics involved [88]. Built on the remarkable advances on the nano-/microparticles and active materials in biomedical imaging, the mobile micro- and nanorobots’ imaging approaches may offer a brand-new active tool for targeting certain areas and executing certain medical tasks in a least invasive fashion [89]. At present, various varieties of micro-/nanorobots have been constructed employing autofluorescence materials for tracking their movements. Traditional photolithography was used to create the first micro- and nanorobots. Using organic compounds with intrinsic fluorescence makes it simple to include fluorescent elements into micro- and nanorobots [90]. Magnetic microrobots and nanorobots can be guided with pinpoint accuracy through complicated biological fluids by means of remote control. Their potential as miniaturized robotic tools for minimally invasive illness diagnosis and treatment stems from their ability to navigate precisely into otherwise inaccessible human body cavities. However, before preclinical in vivo development and clinical trials can begin, crucial challenges, such motion tracking, biocompatibility, biodegradability, and diagnostic/therapeutic effects, must be resolved. Biohybrid magnetic robots with many capacities were described by Yan et al. These robots were created by fusing a biological matrix with an artificial covering to achieve the desired structural and functional characteristics. Superparamagnetic and equipped with powerful navigation capacity in different biofluids, helical microswimmers were produced from Spirulina microalgae using a simple dip-coating procedure in magnetite (Fe_3_O_4_) suspensions [91]. In vivo fluorescent imaging and remote diagnostic sensing were made possible by the microalgae’s inherent characteristics and did not necessitate any surface alteration. As an added bonus, in vivo magnetic resonance imaging was able to follow a group of microswimmers inside the stomachs of rodents, a deep organ where fluorescence-based imaging stopped working due to its lack of penetration. However, depending on the thickness of the Fe_3_O_4_ coating applied during the dip-coating procedure, the microswimmers were able to disintegrate and show specific cytotoxicity towards cancer cell lines. As a proof of concept for the engineering of multifunctional microrobotic and nanorobotic devices, the biohybrid microrobots disclosed here provide a microrobotic platform with potential for further development in in vivo imaging-guided therapy [92]. In vivo imaging applications benefit greatly from autofluorescence imaging due to the technique’s straightforward specificity; nevertheless, its biomedical utility is limited by a number of additional variables. Autofluorescence, caused by fluorescent biomolecules such NAD(P)H, chlorophyll, porphyrins, and collagens, has been shown to permeate key internal organs and body fluids. This finding has implications for the in vivo practical application of fluorescent biomarkers [93]. In addition, the dominant colour of autofluorescence emission is typically either blue or green. Because of its inability to reach deeper layers of tissue, short-wavelength emission light is often reserved for monitoring the skin’s outer layers [94]. Furthermore, with the same excitation light, the various microrobots and living tissue could produce their own unique autofluorescence, leading to significant interference for in vivo imaging. These restrictions limit the clinical applications of autofluorescence microrobots, particularly for deep tissue. This unique technique to the real-time optical visualization of the analyte recognition events was first demonstrated by Jurado-Sánchez et al., who coupled the optical features of QDs with the autonomous movement of artificial nanomachines [95]. Micro- and nanorobots functionalized with quantum dots (QDs) exhibit size-tunable absorption and emission, high fluorescence quantum yields even at NIR wavelengths [96,97,98,99], and huge two-photon action cross-sections, demonstrating superior performance to standard organic dyes. However, there are still some restrictions that need to be dealt with [100]. To begin, the methods for employing QDs have primarily been isolated organic ones. QDs have quite different properties from dye molecules, which make it more difficult to incorporate them into micro- and nanorobots for use in the body. Second, semiconductor quantum dots containing cadmium often raise cytotoxicity concerns before being used in cellular or in vivo research [101].

### 7.3. Magnetic Soft Robotic Grippers

**Figure 8:**
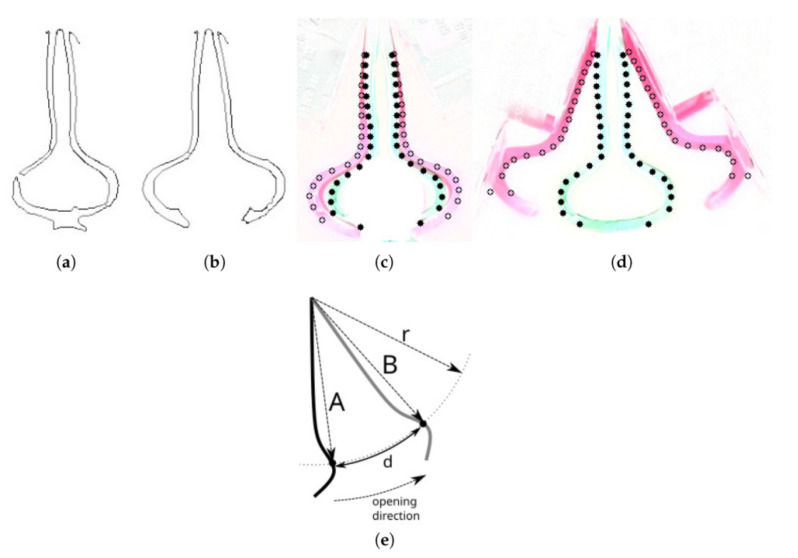
Steps in the preprocessing of the gripper movement analysis. (**a**) Picture of the closed gripper’s edge when the base is closed. (**b**) Picture of the closed gripper holding something on its edge. (**c**) The closed gripper holding an object is different from the closed gripper that is empty (analysis points are marked). (**d**) Picture shows the difference between the closed, empty gripper and the opened gripper, with analysis points marked. (**e**) Finding points of displacement analysis; this is the idea behind it. Reproduced with permission from ref. [108]. © 2022 MDPI.

Animals and robots both require the most fundamental movement of grasping in order to handle objects around them. From the perspective of a materials engineer, Shea and colleagues provide a thorough overview of the state of the art in soft robotic gripper technology, including how it works, how it can be designed, and how it can be fabricated. The section classifies grippers into three distinct categories based on the process by which they grip an object: those that grab by moving their fingers, those that immobilize by changing the stiffness of the envelope around an object, and those that stick by adjusting the adhesion force. Each grasping mechanism has pros and cons with relation to the shape and texture of the object. Here, the actuation of the grabbing fingers, the stiffness of the enclosing MR elastomers and fluids, and the adhesion force between the gripper and the object may all be modulated by an externally generated magnetic field [102]. Since Shea and coworkers have already provided a comprehensive evaluation of magnetic grippers, this section will provide a high-level summary of important post-Shea and coworkers publications on the subject. Significant interest has been focused on developing bionic soft grippers capable of gently conforming to the profiles of objects and holding them with soft, consistent pressure. A unique magneto-active soft gripper has been created elsewhere to realize fast and contactless driving. Actuators called magneto-active elastomers (MAEs) are built into the gripper, and they comprise a soft substrate material and hard magnetic fillers. At first, an external magnetic field is used to magnetize MAE actuators that have been made with varying concentrations of magnetic fillers. The magnetically induced deformation of MAE actuators in response to varying external magnetic fields needs to be measured experimentally. The magneto-active soft gripper was developed after extensive experimental research on MAE actuators [103]. Experimental results show that the magneto-active soft gripper can effectively grasp objects and perform its intended function. Meanwhile, the impacts of applied magnetic field and mass faction of magnetic fillers are investigated, and the maximum gripping force of grippers is measured. When the magnetic field strength or the concentration of magnetic fillers is increased, the gripping force rises; however, once the magnetic field strength is above 80 mT, the gripping force stabilizes. The response time of the magneto-active soft gripper is much shorter than that of existing ones driven by conventional means when performing a grasping motion. The Venus flytrap is an encouraging role model for the advancement of soft robotics due to its high degree of responsiveness and deformability [104,105,106]. One piece demonstrated an innovative robotic gripper with trapping action resembling that of a Venus flytrap. Using a magnetic actuation system and bistable antisymmetric shells, this gripper was built. Two carbon-fibre-reinforced polymer cylinders can be actuated externally to change between two stable configurations, functioning as compliant fingers. The amount of force needed to activate the morphing process can be minimized by imposing a clamped boundary constraint. To actuate the flexible finger, we present a unique noncontact magnetic actuation approach that combines good responsiveness with low power consumption [107]. The robotic gripper is low in mass and size while maintaining a strong grip. The grasping action and actuation force were studied experimentally and computationally. The actuation force required to open a gripper depends primarily on the breadth of the clamped edge. The magnetorheological elastomer shows great potential as a soft robotics material. It is reactive to external magnetic fields and its soft qualities make it possible to create appealing devices. Another work proposed the construction of a soft gripper assembled with magnetorheological elastomers. The magnetorheological elastomers moulding procedure is described in great depth [108]. The magnetorheological elastomers’ electromechanical properties are also demonstrated with a straightforward beam (Figure 8). The soft gripper is then built and tested via a number of trials to determine its effectiveness.

**Figure 9:**
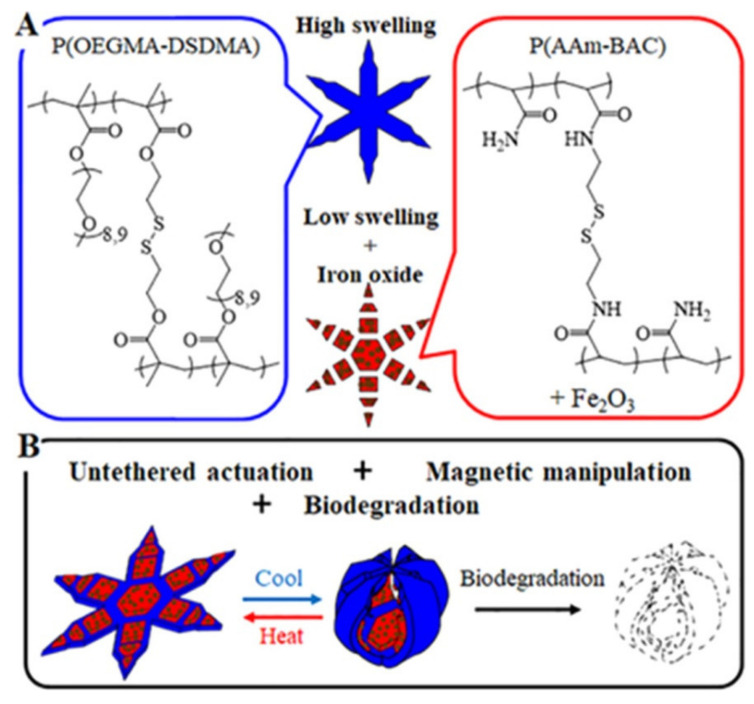
A diagram showing the biodegradable thermomagnetically responsive grippers’ chemical make-up, structure, and how they work. (**A**) The grippers are made up of two layers of hydrogels: a high-swelling P(OEGMA-DSDMA) gel that can be reversed and a low-swelling P(AAm-BAC) gel. The P(AAm-BAC) gel also has magnetic Fe_2_O_3_ NPs added to it. (**B**) An example of how the soft grippers can be used for more than one thing, such as biodegradation, magnetic handling, and thermally responsive actuation. Reproduced with permission from ref. [117]. © 2019 American Chemical Society.

There is a growing trend towards making robotic grippers smaller because it is necessary to move small items precisely in small spaces, but there are a lot of problems related to microfabricating, putting together, and moving the grippers that are getting smaller and smaller. To get around these problems, a group of experts shared a way to 3D print a triple-finger microgripper that is powered by magnets and can be used for strong micromanipulation in both air and water. Magnetic control was chosen because it allows operation without a cord in complicated environments. Using the microcontinuous liquid interface production (μCLIP) process, a single-piece microgripper design with flexible mechanical parts and magnetic force actuation units was quickly made. This reduced the trade-off between mechanical compliance and magnetic actuation forces. Finally, we connected the 3D-printed gripper to a robot arm and showed that it could move tiny items in water and air [109]. This work could lead to biological and medical uses, like being able to work on living cells and soft organs. Ferromagnetic materials can be precisely and deftly manipulated in real time thanks to the presence of magnetic fields. Glass, organics, and metals are examples of materials that do not respond to a magnetic field because they are not magnetic. In order to manipulate nonmagnetic objects magnetically, a transitional ferrofluid (TF) is introduced here. TFs are created by encasing magnetic iron particles in pure gallium using highly concentrated HCl solutions, and their interlocking force on objects can be toggled during the phase change [110]. A liquid TF gripper may make close contact with an object of any shape and then solidify at room temperature to generate an interlocking force of up to 1168 N (with just 10 g TF), which can be easily dissipated (F 0.01 N) by melting. A solid TF can be melted by electromagnetic induction heating due to its high electrical conductivity and magnetism. Embedded nonmagnetic items can be influenced by an applied magnetic field thanks to the TF’s switchable physical force during the phase transition and magnetism, and then they can revert to being immune to magnetic stimuli after the TF has been heated and released. Polymeric microgrippers are an example of a soft-robotic device that could be used in medication delivery, minimally invasive surgery, or biomedical engineering to pick up and insert delicate biological cargo in inaccessible conduits [102,111,112]. Self-folding thermomagnetically responsive soft grippers on the millimetre scale have been designed, built, and used for pick-and-place applications; however, it is worrying that such tiny devices might be forgotten or left behind after their use in actual clinical applications [113,114,115,116]. Consequently, measures need to be devised to ensure that these soft robotic devices are biodegradable so that they would disintegrate if left behind in the body. Kobayashi et al. reported the photopatterning of bilayer gels consisting of a thermally responsive high-swelling poly(oligoethylene glycol methyl ether methacrylate and a low-swelling poly(acrylamide-N,N′-bis(acyloyl)cys. The temperature-induced swelling of the P(OEGMA-DSDMA) layer allows the grippers to alter shape in response to thermal stimuli, allowing them to open and close. They also showed that the grippers may be loaded with substances for prospective uses as drug-eluting theragrippers by doping them with magnetic nanoparticles. The reduction breakage of disulfide links also makes them biodegradable at body temperature (37 °C) [117]. Combining thermoresponsive shape change with magnetic navigation and biodegradability is a major step forward in ensuring the secure use of shape-changing (Figure 9), untethered biomedical devices and soft robots in surgical and medical situations.

By modulating the force of attraction between the gripper’s pedal and the object, magnetic fields have also been used to realize pick-and-place handling. A cylindrical composite pad created by Gong and colleagues has an MR gel core and a PDMS exterior [118]. In this research, dry adhesion is achievable since the pad is in such close proximity to the object. The composite pad’s PDMS surface is so soft and viscoelastic that it may “flow” into nanoscopic roughness in the object’s surface, thus maximizing the close contact. But when a magnetic field is introduced, the MR gel core within the material stiffens and deforms, disrupting the close contact and causing a local separation (what is commonly called a “crack” in adhesion research). Viscoelastic adhesion is also avoided by the composite pad due to its higher stiffness.

## 8. Conclusions and Outlook

Magnetic fields offer an unrivalled advantage over other stimulation sources when it comes to penetrating deeply into a variety of materials, allowing them to activate the actuation of soft robotics. Furthermore, magnetically susceptible materials can react rapidly to varying magnetic field strengths. It is possible to generate a precisely tunable spatial gradient inside a confined container using an array of numerous electromagnets or a set of revolving permanent magnets. In light of these considerations, it can be concluded that using a magnetic field to actuate soft robotic devices is a very desirable alternative for a wide range of biomedical applications, such as minimally invasive surgery and precision medication delivery. The initial step in successfully applying these medical devices when untethered is to regulate their swimming and terrestrial locomotions with an applied magnetic field. Gripping is the most fundamental and vital method of motion for both free-floating and attached devices to realize virtually any practical application. The use of magnetic elastomers and gels as magnetic soft actuators has the potential to revolutionize the area of magnetically controlled soft robotics by maximizing the benefits that soft robotics can bring to the table. Elastomers and gels have mechanical qualities similar to rubber and may be formed into almost any shape. In addition, the magnetic soft actuator’s motion can be accurately programmed with the help of controlled magnetization, which involves applying a magnetic field with patterned control. Two of the most important ideas for achieving complicated programmable motions are highlighted in this review: constantly distributed magnetization and programmed magnetic axis alignment techniques.

Recent breakthroughs in soft material production technologies like 3D printing, origami/kirigami, robust hydrogels, mechanical metamaterials, and liquid metal-injected elastomers have led to rapid advancements in the field of soft actuators during the past decade. There have been several promising developments in magnetic soft actuator materials prompted by the new wave of material technologies. However, some of these developments in materials technology have not yet been implemented into soft robotics systems. The use of magnetic soft actuators in soft robotic devices is justifiable due to their many advantages, notably in biomedical applications, despite the fact that elastomers and gels present some inherent difficulties that make precisely regulated actuation difficult. When precise control is needed, the successful model can encourage robotics developers to incorporate magnetic elastomers and gels in their design. All of these developments have the potential to affect the related field of soft sensors. In summary, the disciplines of magnetic soft actuators and soft robotics are fast-growing due to recent advancements in materials engineering, and the potential of these innovative materials is just beginning to emerge.

## Figures and Tables

**Table 1:** A comparison of common ways to build and programme robots and magnetic soft materials.

Method	Matrix	Magnetic Component	Shape	Resolution	Ref.
Molding	Silicone	NdFeB	2D	~0.5 mm	[54,55]
Extrusion 3D printing	Silicone	NdFeB	3D	~100 µm	[48]
Digital light processing	Photocurable elastomer	SPIONs	2D	~100 µm	[53]
Micromoulding	Silicone	Cobalt nanomagnet	3D	~50 µm	[56]
UV photolithography	Hydrogel	SPIONs	2D	~1.5 µm	[57,58,59]
Electron beam lithography	Flexible silicone thin film	Cobalt nanomagnet	2D	~0.1 µm	[60]

## Data Availability

Data are contained within the article.
